# Genomic perspectives on the evolution and spread of bacterial pathogens

**DOI:** 10.1098/rspb.2015.0488

**Published:** 2015-12-22

**Authors:** Stephen D. Bentley, Julian Parkhill

**Affiliations:** The Sanger Institute, Wellcome Trust Genome Campus, Hinxton, Cambridge CB10 1SA, UK

**Keywords:** bacterial evolution, bacterial transmission, bacterial genomics

## Abstract

Since the first complete sequencing of a free-living organism, *Haemophilus influenzae*, genomics has been used to probe both the biology of bacterial pathogens and their evolution. Single-genome approaches provided information on the repertoire of virulence determinants and host-interaction factors, and, along with comparative analyses, allowed the proposal of hypotheses to explain the evolution of many of these traits. These analyses suggested many bacterial pathogens to be of relatively recent origin and identified genome degradation as a key aspect of host adaptation. The advent of very-high-throughput sequencing has allowed for detailed phylogenetic analysis of many important pathogens, revealing patterns of global and local spread, and recent evolution in response to pressure from therapeutics and the human immune system. Such analyses have shown that bacteria can evolve and transmit very rapidly, with emerging clones showing adaptation and global spread over years or decades. The resolution achieved with whole-genome sequencing has shown considerable benefits in clinical microbiology, enabling accurate outbreak tracking within hospitals and across continents. Continued large-scale sequencing promises many further insights into genetic determinants of drug resistance, virulence and transmission in bacterial pathogens.

## 1. Introduction

Formal characterization of disease-causing agents has always been fundamental to understanding the evolution of that pathogen and the epidemiology of infectious disease. The ability to identify similarities and differences in character among pathogen isolates enables their separation into groups or ‘types’. Linking this information with temporal, spatial and clinical data can bring understanding of evolution, geographical spread and disease associations for the pathogen, providing vital information for identifying sources of infection and for designing interventions to prevent and treat disease.

For bacterial pathogens, the array of methods for ‘typing’ isolates is vast and includes techniques for detecting differences in biochemical activity, protein production, toxin production, susceptibility to bacteriophage infection, susceptibility to the action of antibiotics and reactivity with antigen-specific sera. These methods have proved to be very valuable, leading to the establishment of globally accepted typing schemes as the standard to characterize the members of specific bacterial species. However, there are several drawbacks: (i) there is little interoperability of typing schemes between species, making it difficult to compare the behaviour of different pathogens; (ii) the expression of the detected characteristic by an individual isolate can vary, potentially leading to false-negative results; (iii) interpretation of the assay result can be highly subjective (most notably for serological tests), potentially leading to false-positive and false-negative results, and variation between testing institutions; (iv) the genes encoding the detected characteristic can be gained or lost through horizontal gene transfer, potentially giving a false impression of relatedness between isolates.

To capture the true ancestral relationships between isolates, one needs to study variation in the heritable material, DNA. Progress in DNA sequencing has brought a steady accumulation of bacterial gene sequence information that has allowed the development of typing schemes that exploit variation in these sequences [1]. Some schemes focus on single genes or loci with sufficient variation to provide distinguishing data but are often susceptible to misleading designations due to horizontal gene transfer and strong selective pressures that can lead to anomalies in the patterns of sequence variation. A more robust approach is that of multilocus sequence typing (MLST), which aims to capture the sequence variation at multiple loci (usually six to eight) distributed around the chromosome [2]. The loci selected for MLST encode housekeeping functions, so are not likely to be under strong selective pressure and should vary in a clock-like manner, thus giving a good signal of ancestral relationships. The unifying nature of DNA means that MLST analysis findings can be compared between species and recombination of loci between strains within a species can be detected. MLST has allowed the unambiguous identification of clones within pathogen species that have particular associations with disease, enabling robust systems for the tracking of the spread of such clones, but limits of resolution mean that differentiating between members of a clone is often not possible, and the exact details of transmission paths therefore remain obscure.

Whole-genome sequence (WGS) data can provide a complete picture of gene repertoire and sequence variation. The first whole bacterial genome sequences largely comprised single representatives of major pathogen species and were generated using first generation sequencing technologies, essentially based on the methods developed by Fred Sanger and colleagues in the 1970s [3–6]. Comparison of these reference pathogen genomes with those from closely related pathogenic and non-pathogenic species brought dramatic advances in understanding of pathogen evolution and host adaptation. The second generation of sequencing technologies, which emerged around 2008, delivers much more data at lower costs [7]. Accordingly, they have increased the availability of WGS data and allowed the sequencing of hundreds or thousands of isolates from distinct populations of bacteria. These data produce fine resolution of gene and sequence variation, empowering the study of evolutionary mechanisms, phylogenies and transmission events, and have created a further wave of discovery in our understanding of pathogen biology. The emerging third generation of sequencing technologies promises further advances with the potential for real-time tracking of outbreaks and the ability to capture epigenetic changes that may affect pathogen behaviour.

The purpose of this review is to highlight the areas where genomic analyses have provided new insights and to demonstrate their potential for unravelling the origin, transmission and evolution of bacterial pathogens.

## 2. Host adaptation and evolution of pathogenesis

In the early days of expensive, highly polished, single genomes, information about evolutionary pathways and pathogenicity had to be gleaned from effectively few observations and sparse comparisons across large evolutionary distances. Nonetheless, big themes did emerge from these analyses. Genome sequences of bacteria allowed the complete cataloguing of host-interaction and pathogenicity genes within individual pathogens, often identifying unknown systems and generating hypotheses for experimental analysis. Bacterial genetics had already identified multiple mechanisms of horizontal transfer of DNA between bacteria, and mobile elements such as bacteriophage, plasmids and pathogenicity islands were known to carry genes involved in pathogenicity and drug resistance and transmit them between bacteria. Such mobile elements could be identified within genomes from signatures such as anomalous nucleotide composition and the presence of signature mobility genes, showing that many genomes had significant amounts of DNA of apparently horizontal origin, but of unknown age [8]. The first pairwise comparisons within species, for example the comparison between the laboratory strain *Escherichia coli* K12, and the pathogenic *E. coli* O157 : H7 [9], revealed that a very large number of genes (26% in this case) could be unique in the pathogen compared with the non-pathogen. Increasing numbers of within-species comparisons built up to perhaps the most surprising early observation from comparative analysis: the incredible diversity found within individual bacterial species in terms of gene presence/absence, with individual isolates containing very large numbers of unique genes, and an increasingly small number of genes present in all members of any one species. This was formalized into the concept of the pan-genome: that bacterial species have core genes found in every member, and accessory genes found in a subset of strains that code for strain-specific adaptations, often pathogenicity-related, with the pan-genome representing all of the genes present in the species [10,11].

Against this diversity, some pathogens are clearly monomorphic, with little, if any, mobile DNA, and very little nucleotide diversity. These monomorphic lineages include key human pathogens such as *Yersinia pestis* (plague) [12], *Bordetella pertussis* (whooping cough) [13] and *Salmonella typhi* (typhoid fever) [14]. Analysis of the genomes of such pathogens identified a set of common (and surprising) features. Prior to the advent of genomics, it was a frequent assumption that bacterial genomes would be streamlined, carrying little, if any, non-functional DNA. However, many of these monomorphic pathogens challenged this assumption in two ways. First, they often had large expansions of repetitive, selfish DNA in the form of insertion sequences. Second, they often had large numbers of pseudogenes—genes inactivated by point mutation or disruption. Even more curiously, these pseudogenes were frequently inactivated genes previously involved in pathogenicity or host interactions, despite these being virulent pathogens. Another factor common to many of these pathogens helped explain these phenomena—many of these pathogens were host-restricted descendants of previously broad-host-range ancestors. This suggested a common evolutionary pathway. These organisms had invaded a new niche, in the case of *S. typhi* and *Y. pestis*, moving from gastrointestinal to systemic disease, and this was often enabled by the acquisition of novel pathogenicity genes on mobile elements (on plasmids for *Y. pestis*, and on chromosomal mobile elements for *S. typhi*) [12,14]. This was accompanied by loss of genes required for the previous niche, but also of genes that were disadvantageous in the new niche. Not all of the gene loss could be ascribed to selected changes, however, and it was hypothesized that many were due to accelerated mutation fixation due to the evolutionary bottleneck associated with niche change, a process that would also explain the expansion of selfish mobile elements in the chromosome. *Bordetella pertussis* manifested an extreme example of this, where the niche change, host restriction and increase in virulence appeared to have occurred purely by genome degradation, without any acquisition of novel genes [13,15].

A similar pathway was evident in some animal pathogens (such as *Burkholderia mallei*, which causes glanders in horses [16]) and plant pathogens (for example *Xanthomonas oryzae*, a pathogen of rice [17]). One common factor linking all of these different evolutionary transitions is the Neolithic revolution, in which the human population increased in size, and human settlements became denser and more permanent, creating a new niche for human pathogens. At the same time, humans created large monocultures of crop plants and farm animals, creating similar novel niches for these pathogens [18].

The ability to sequence single genomes provided a wealth of information on pathogenicity and its evolution over long time scales. However, the expense and difficulty of generating genomes with clone-based, Sanger-sequencing technology limited the ability to look at the fine-scale changes that would inform on recent evolution and spread. The only real attempt to do this with older technology formed part of the investigation into the anthrax attacks in America in 2001 [19], where nearly a dozen genomes were generated at significant expense to help identify the source of the strain used.

## 3. Advances in sequencing technologies open up new areas of analysis

The large-scale sequencing required to investigate fine-scale evolution and transmission in bacterial pathogens required a move away from clone-based sequencing to the highly parallel, clone-free approaches currently in use. Over the five or so years of this transition, costs for sequencing bacterial genomes dropped from tens of thousands of pounds to tens of pounds, and the time taken from months to days, or hours. This drop in costs enables large collections of near-identical strains to be sequenced, giving the potential for very-high-resolution identification of the recent ancestral relationships between isolates and, by linking this with epidemiological data, the true patterns of spread. The first datasets generated using these approaches for the first time allowed sequencing of isolates sampled from across the majority of the age of a bacterial lineage, and this revealed a very surprising finding. The substitution rate (the rate of fixation of mutations in the population) was at least a hundredfold higher than previous estimates which had been used to date recent clonal pathogens. Most of this dating was based on an estimate of the neutral mutation rate since the divergence of *E. coli* and *Salmonella typhimurium*, of around 6–8 × 10^−9^ per site per year [20,21]. The first estimate based on large-scale sequencing of isolates within a recent clone, *Staphylococcus aureus* ST239 [22], produced an estimate of 3.3 × 10^−6^ per site per year. Similar rates have been identified in *Streptococcus pneumoniae* [23], *Vibrio cholerae* [24], *E. coli* [25] and others. The consequences of this are profound. It means that, contrary to expectation, bacteria constitute a measurably evolving population, and that phylogenetic analysis tools developed to analyse the populations of rapidly evolving viruses can be used to analyse bacteria, allowing detailed descriptions of geographical and temporal transitions on global and local scales, and giving sufficient resolution, in many cases, to track pathogen transmission events between individual patients in a hospital. Often the conclusions confirm speculation from lower-resolution typing techniques, but with greater clarity and with the potential to reveal and explain deeper epidemiological patterns and precise transmission events.

## 4. Global spread of bacterial pathogens

Bacterial pathogens can be broadly categorized based on the nature of their host association, ranging from environmental organisms that occasionally and accidentally infect, through common commensal colonizers that infect sporadically, to obligate pathogens that do not exist outside of the host. In all cases, and to varying degrees, the global spread of pathogenic clones is observed; genome analysis has the capacity to clarify the patterns of spread and highlight characteristics that may influence spreading potential.

Cholera outbreaks have long been a scourge of mankind, with some archeological suggestions of cholera-like illness in the plains of the Ganges River since ancient times [26]. Cholera disease outside of the Bay of Bengal is typically characterized by intermittent and unpredictable outbreaks followed by regional decline. The epidemic dynamics remain poorly understood, but population genomic studies have brought significant new understanding of the evolution and long-range spread of the causative agent, *V. cholerae*. Historically, cholera is described as occurring in seven global pandemics, with the first starting in southeast Asia in 1817 and the current pandemic having started in Indonesia in 1961. Genomic analysis showed that the El Tor lineage responsible for the seventh pandemic had independently emerged from environmental *V. cholerae*, separate from the Classical lineage responsible for the first six [27]. Deeper analysis of 154 isolates collected across the temporal and geographical breadth of the seventh pandemic allowed construction of a robust phylogeny of the El Tor lineage by identifying single-nucleotide variations which define the ancestral relationship between isolates [24]. Combining the phylogeny with the time and place of isolation for each isolate showed that the seventh pandemic clone had emerged in the region of the Bay of Bengal and spread from there to the rest of the world. Furthermore, it was shown that the seventh pandemic actually comprised at least three separate waves, with second and third waves derived through ongoing evolution of the *V. cholerae* population within the Bay of Bengal region, with acquisition of mobile genetic elements playing a key role.

The detailed phylogeographic structure of these data can greatly inform understanding of contemporary outbreaks. For example, the devastating cholera outbreak that began in Haiti in 2010 was highly controversial, with conflicting hypotheses regarding the source of the epidemic. Suggestions for the source included expansion of local Haitian strains, import from the Mexican Gulf during the recent earthquake and import from more distant countries by human carriage. WGS analysis of isolates from the outbreak, alongside WGS data for isolates from around the globe, was able to show clear results consistent with Nepal as the origin of the Haitian outbreak [28,29]. Haiti had not had a cholera outbreak in over 100 years, and these findings highlight how local microbiology can be dramatically changed and how infectious agents can be transmitted globally through international travel. Further genomics-based analysis of Haitian isolates has used phylogenetics to show the last common ancestor of these strains temporally overlaps with the date of introduction and has shown how this introduced strain has evolved locally after being introduced [30–32].

Most of the evolution for the species *V. cholerae* occurs in its aquatic environmental niche from where it can be isolated in all parts of the world. There is therefore a near ubiquitous source for cholera infection, should the appropriate conditions occur, but the majority of outbreaks are due to a succession of clones which have evolved around the Bay of Bengal. *Pseudomonas aeruginosa* is a common opportunistic pathogen of humans that also has a primarily environmental niche which shapes the species's evolution [33,34]. The major disease burden of *P*. *aeruginosa* is in morbidity and mortality of lung infections in cystic fibrosis (CF) sufferers. WGS analysis has been applied to study the relationships between isolates from infections and environmental samples [35]. The study based in Ontario, Canada, showed that the breadth of genetic diversity of isolates collected from CF sufferers is broadly equivalent to that seen from environmental sampling, confirming the environment as a major source of infection, but also showed that within the broad diversity, there was an expansion of two highly prevalent ‘epidemic’ clones in the disease-associated population. Isolates in one of the identified epidemic clones were almost identical to an isolate representative of the Liverpool epidemic strain which is frequently isolated from CF lung infections in the United Kingdom, giving strong evidence of intercontinental transmission, most likely via contacts between individuals within the CF community. This suggests the emergence of clones specifically adapted to the CF lung environment, a phenomenon that has also been identified for two other CF-associated pathogens, *Burkholderia cenocepacia* [36] and *Mycobacterium abscessus* [37], using pre-genomic and genomic approaches, respectively.

Some major bacterial pathogen species can be considered as globally endemic with regional variation in disease rates correlated with such parameters as nutrition and economic status. These include so-called commensal respiratory pathogens, such as *S. pneumoniae*, *Neisseria meningitidis* and *Haemophilus influenzae*, that are niche-restricted to the human upper respiratory surfaces and are thus in constant balancing evolution with the human immune system. This balance may be maintained through frequent transmission between hosts and frequent variation in surface antigens, largely through horizontal gene exchange. With multiple lineages coexisting, these populations are locally dynamic, but there is also a tendency for emergence of pandemic clones that appear to have an advantage in colonization and transmission, and are seen to spread globally [38]. However, the epidemiological study of these populations can be confounded by their high rates of homologous recombination.

The *S. pneumoniae* clone known as PMEN1 was the first recognized pandemic pneumococcal lineage. It was seen to emerge in Spain in the 1980s as a multidrug-resistant serotype 23F, was subsequently identified in Africa, Asia and America, and, by the late 1990s, was estimated to be causing almost 40% of penicillin-resistant pneumococcal disease in the USA [39]. WGS analysis of 240 PMEN1 isolates collected from 22 countries and four continents over the period 1984–2008 revealed a detailed phylogeny supporting a European origin for the clone, with further sub-clones developing within that continent [23]. Other sub-clones rooted within the European clones indicate multiple discrete, successful intercontinental transmission events indicated by tight clusters associated with South Africa, Vietnam, USA, and Central and South America. Most strikingly, the PMEN1 phylogeny indicates the emergence of a sub-clone that appears to have spread more rapidly between continents and thus seems to have an enhanced ability to spread, as compared with the PMEN1 clone as a whole. The genetic basis of this enhanced spreading capacity is unclear but emerging datasets for different clones of varying spreading capacity should provide the data to allow discovery of such genetic associations [40–42].

WGS analysis of other pneumococcal clones has highlighted differing characteristics and identified possible genetic explanations for differing patterns of spread. Clone PMEN2 emerged from a beta-lactam susceptible clone, which gave rise to two resistant clones through independent acquisition events—one clone, PMEN22, was never observed to have spread beyond Europe while the other, PMEN2, expanded in Europe and spawned multiple intercontinental transmissions, including one to North America which went on to transmit to southeast Asia and the Middle East [41]. PMEN2 transmitted to Iceland, where it rose to high prevalence and dominated drug-resistant infections. Phylogenomic analysis showed that the clone had been introduced to Iceland in two separate events, but that only one prevailed and expanded. Interestingly, the analysis showed that, while establishing in Iceland, the clone had acquired a mutation that prevented further recombination. This would have reduced the ability of the clone to diversify and may have been a significant factor in its subsequent rapid decline.

*Staphylococcus aureus* is another bacterial pathogen frequently seen as a colonizer of healthy humans, most commonly in the nares and on the skin. Many of the globally spreading clones are those that appear to have adapted to colonization in hospital settings, where they cause major problems through infections associated with surgery, invasive lines and wounds in immunosuppressed individuals. As was seen for the pneumococcal clone PMEN1, the first globally dominant clone of hospital-associated *S. aureus* (ST239) was seen to emerge in southwestern Europe and spread globally through multiple international transmissions [22]. Phylogenomic analysis, linked with epidemiological data, indicated that the clone had been transmitted from the Iberian Peninsula to South America in the early 1990s. In the following years, the clone declined in prevalence in Portugal but was seen to re-emerge in the late 1990s. The genomic data showed that this re-emergence was likely to have been due to transmission of the clone back from South America to Europe. The resolution of the ST239 phylogeny was also able to identify distinct recent transmissions from southeast Asia to Europe (one to Denmark and another to the UK). Similar analysis of the *S. aureus* ST22 clone, which had emerged in the UK in the early 1990s and is currently spreading globally, was able to show that this hospital-associated clone had emerged from a community-associated ancestor [43], and that this emergence was apparently not due to methicillin resistance, but may have been associated with resistance to fluoroquinolones. Genomic analyses also implicated hospital adaptation in the emergence of other epidemic clones of methicillin-resistant *S. aureus* (MRSA) such as CC30 [44], and delineated transmission between animal and human hosts in the livestock-associated CC398 [45].

Many bacterial pathogens where significant global spread is detected tend to move through human-to-human transmission and this appears to happen sufficiently frequently that successions mask earlier clones. For *Mycobacterium tuberculosis*, the evolutionary landscape is somewhat distinct from this, with apparently slower rates of evolution probably due to low rates of replication. Pre-genomic typing techniques distinguished a handful of *M. tuberculosis* clones and demonstrated a close relationship with an animal-associated species (*Mycobacterium bovis*). Early dogma therefore suggested that human tuberculosis was a disease that had been acquired zoonotically, probably as a result of domestication. However, patterns of large chromosomal deletions, many determined from WGSs, have been used in phylogenetic reconstructions to provide strong evidence that *M. bovis* actually emerged from a *M. tuberculosis*-like ancestor [46], so the transmission is more likely to have been from human to animal. Subsequent genomic analyses have been able to add a temporal perspective, leading to the proposal that *M. tuberculosis* first infected humans in Africa around 70 000 years ago and spread globally with the migration of humans out of Africa [47]. The study was based on similarities in phylogenetic tree topology between the branching structure and geographical distribution of a genomic tree constructed for a global collection of 220 *M. tuberculosis* isolates, and that of a tree of human lineages constructed from nearly 5000 mitochondrial DNA sequences. A Bayesian statistical method was applied to test whether the branching points of the *M. tuberculosis* phylogeny correlated with major points in the history of modern humans, identifying similarities in the spread of TB and humans into Asia and Europe. However, convincing as this scenario is, it is contradicted by more recent evidence using ancient DNA samples from South America to date the most recent common ancestor of the *M. tuberculosis* complex to less than 6000 years ago, meaning that TB cannot have spread as proposed, and that the first introduction into the New World may have been via marine mammals, and not human migrations [48].

## 5. Using genomics to study local transmissions and outbreaks

Outbreaks of specific pathogens can occur either through single sources (e.g. food-borne outbreaks) or through increased transmission of a single infective agent. This is most often observed on a local level within a defined community, geographical area or season, but may extend to larger areas, and even globally. The study of infectious disease outbreaks can be greatly enhanced by WGS analysis of relevant pathogen isolates, potentially providing a strong indication of the primary source of infection and, where coupled with sufficient epidemiological information, indicating the routes of onward transmission. The three parameters most crucial to this work are the high-resolution genotype provided by WGS data, the time of isolation of the pathogen sample and the location of the individual from whom the sample was derived. Outbreaks that have been studied thus far using WGS analysis can be grouped into three categories of infection route: food-borne transmission, healthcare-associated acquisition and environmental transmission.

Food-borne outbreaks tend to focus on identification of the causal infectious agent followed by a search for the source of that agent within food production and supply pipelines. During May and June of 2011 in Germany, an outbreak of more than 3000 cases of infection by Shiga-toxin-producing *E. coli* O104 : H4 was reported [49]. This outbreak was notable and unusual in the numbers of infections and the severity of disease; around 25% of infections involved hemolytic = uremic syndrome, while rates of only 1–15% had been seen in previous outbreaks involving Shiga-toxin-producing *E. coli*. Also notable was the rapid availability of WGS data driven by public release of second- and third-generation sequencing technology data and a major ‘crowd-sourced’ analysis endeavour involving an international cohort of bioinformatics experts [50]. Although the analysis could not fully explain the high degree of virulence seen in the outbreak, it did identify acquisition of gene clusters that could contribute to the nature of the outbreak, including genes for production of toxin and genes for surface structures that could affect efficiency of adherence to human tissue or food material. The source of infection for the outbreak in Germany was identified as bean sprouts, using epidemiological and microbiological data, so no further genomic analysis was required. However, subsequent cases of infection due to Shiga-toxin-producing *E. coli* in France and Turkey raised concern that the outbreak could be ongoing and more widespread. Here, the resolving power of genome data was able to demonstrate that these new cases were caused by unrelated strains, so not part of the same outbreak, but also highlighted the potential for future outbreaks from strains with similar virulence characteristics [51]. WGS is now being developed for routine use in the source attribution of food-borne pathogens, due to its superior resolution over conventional typing techniques [52,53].

The overall increased susceptibility of hospital populations heightens the risk of acquisition of bacterial infections that tend to be dominated by specific clones of a few species (e.g. MRSA *Clostridium difficile*, vancomycin-resistant *Enterococcus* and carbapenem-resistant Enterobacteriaceae) that appear well adapted to the hospital environment. Members of these adapted clones tend to be indistinguishable using conventional typing techniques, making it difficult to detect outbreaks and transmissions. For MRSA, the nosocomial infection rates in many European countries and the negative impact of infection on outcome have led to a ‘zero tolerance’ for colonization, with many hospitals routinely testing for colonization for all admitted patients. One of the first bacterial population genomic studies showed that WGS data could provide sufficient resolution to differentiate MRSA isolates of the same clone, from the same hospital ward, separated by a short period of time [22]. Further studies showed that, when combined with epidemiological data, this resolution could be used to reconstruct a hospital outbreak of MRSA transmission, discovering onward transmission into the community and identifying a hospital worker involved in maintaining the outbreak [54]. Other analyses have shown that WGS can be used to track transmission within hospitals of important nosocomial pathogens, such as *Klebsiella pneumoniae* [55] or *Acinetobacter baumanii* [56]. Despite these successes, deeper analysis has raised concerns that interpretation of transmission data could be challenged by the existence of genomic diversity within a colonized individual, combined with the limitation of single colony purification in microbiological practice, and the uncertainty of the size of transmission bottleneck size [57]. Such within-host diversity has been demonstrated in carriage of *S. aureus* [58], and in MRSA on hospital wards in a high-transmission setting [59]. Understanding the dynamics and transmission of this diversity will be important in future use of WGS for hospital outbreak analysis.

Analysis may be further complicated for spore-forming pathogens such as *Clostridium difficile*, where the time periods and rates of mutation in this dormant state are poorly defined. WGS analysis was used to characterize isolates from all hospital and community-associated cases of *C. difficile* over 3.6 years in Oxfordshire, UK, to inform study of transmission dynamics. Evolutionary modelling was applied to define relatedness of individual isolates and to assign single-nucleotide polymorphism (SNP) thresholds for designating samples within or outside of a transmission [60]. This approach determined that only around one-third of all cases were due to transmission from a symptomatic individual. The remainder were concluded to be due to an unsampled reservoir of genetically diverse sources that could include asymptomatic carriers and the local physical environment. Importantly, the analysis was able to detect likely transmission events where there was no supporting epidemiological data available providing direction for focused investigations to discover novel routes of infection.

*Mycobacterium tuberculosis* also presents challenges for data interpretation as its life cycle includes periods of latency when it is assumed that generation of sequence diversity through mutation is slowed. However, efforts in genomic epidemiology of tuberculosis outbreaks have led to progress. A retrospective study of samples from cases of TB collected across the UK Midlands region between 1994 and 2011 analysed WGS data from 390 isolates sampled from 254 TB patients [61]. Again, SNP thresholds were used to define transmission links, with more than 12 SNPs indicating that isolates were not transmission-linked. For the 114 transmission pairs identified using genetic and epidemiological data, 96% were separated by fewer than five SNPs. These approaches can also be used to differentiate between re-infection and recrudescence in individuals within an endemic environment [62,63]. After successful treatment, individuals can present with a new infection, which can be due to either re-infection through transmission or re-activation of a latent infection that effectively represents a treatment failure. Differentiating these states is often only possible with the resolution available through WGS, and this provides vital information for the management of infection control interventions. The resolution of genome-based epidemiology has also been successful in advancing the understanding of the causes of local TB outbreaks. A study focused on a TB outbreak in Vancouver, Canada, combined genomic data with contact tracing information to try to explain the sudden upsurge in cases [64]. The genomic analysis determined that there had been two separate lineages of the same MIRU-VNTR type circulating, with both lineages contributing to a transmission network including multiple ‘superspreaders’ who initiated many of the infections. The outbreak was associated with drug misuse, and the analysis showed that the trigger for the outbreak was likely to be due to an increase in the availability of crack cocaine rather than any genetic change in the pathogen.

*Legionella pneumophila*, the causative agent of Legionnaire's disease, is an environmental bacterium associated with sporadic outbreaks of severe pneumonia [65]. Legionnaire's disease outbreak investigation is normally limited to epidemiological analysis of patient movements prior to illness, and the source of infection is often attributed to air conditioning units of large buildings or domestic and industrial water sources, such as hot tubs and food processing plants [66]. Rates of positive bacterial culture for *L*. *pneumophila* are low (15–20%) and pathogen genetic analysis has usually been limited to PCR typing, so detailed transmission analysis is normally unfeasible. An outbreak of Legionnaire's disease in Edinburgh, Scotland, in 2012 yielded positive cultures from 15 of 91 infected individuals, including multiple isolates from some. Phylogenomic analysis showed levels of diversity that could not have been generated during the course of the outbreak, suggesting that most infections were due to multiple related lineages that had developed in an unknown environmental niche [67]. Notably, one individual was shown to be infected with two unrelated lineages of *L*. *pneumophila*, indicating that the source of infection harboured a diverse population.

Clearly, epidemiological data are crucial for explaining outbreaks and WGS data alone cannot be relied upon to determine likely transmissions. Furthermore, analysis of WGS data from outbreak samples should not be performed without comparison with non-outbreak reference data. Both of these points were emphasized by the investigation of an outbreak of tularaemia that occurred in Sweden in the summer of 2010 involving 67 cases, the largest outbreak seen globally since 1967 [68]. The causal agent, *Francisella tularensis*, is an environmental host-dependent pathogen resident in a variety of animals and transmitted by insect vectors, though it can also survive outside of the host and disperse through aerosols. WGS analysis of 10 of the Swedish outbreak isolates showed maximum pairwise distances between isolates of up to 15 SNPs, which, taken alone, would be consistent with a single-source outbreak. However, the geographical separation and timing of the cases made this doubtful; when compared with a global collection of reference genomes, outbreak isolates were often found to be more closely related to the reference strains than other outbreak isolates. Overall, the analysis indicated that *F*. *tularensis* has a slow mutation rate that may be explained by the life cycle with an intracellular and environmental-dormant phase. As with the Vancouver TB outbreak, the trigger for the outbreak was non-genetic and is better explained by seasonal ecological change.

## 6. Perspectives

Genomics has allowed a significant advance in our understanding of the evolution and spread of bacterial pathogens. Early, single-genome approaches provided information on the unanticipated diversity of bacterial genomes, as well as identifying pathogenicity determinants and outlining the evolutionary trajectories taken by many key bacterial pathogens. Large-scale whole-genome sequencing has elucidated in precise detail the transmission pathways of pathogens at a global and local level. The future will undoubtedly involve routine WGS in clinical microbiology, both for tracking transmission and spread of pathogens, as well as prediction of drug-resistance profiles, based on these advances [69,70]. On the research side, the increasing availability of genomes from large, well-phenotyped collections will allow the identification of genetic determinants of many pathogenicity-associated phenotypes using the genome-wide association approaches that have been so successful in human genetics. Early studies using phenotypes such as host range and drug resistance have already begun to bear fruit [71–73]. New genomic technologies will be likely to reduce the time and sample preparation needed for sequencing, allowing real-time and near-patient sequencing for rapid outbreak detection and analysis of evolution as it occurs in the wild [74]. Continuing decreases in cost will allow whole-genome approaches to the study of experimental evolution, radically expanding our understanding of evolutionary mechanisms [75]. At the same time, single-molecule sequencing technology is allowing DNA modifications to be assayed directly, with recent results uncovering arguably the first global epigenetic regulatory system in a bacterium [76]. Many more novelties almost certainly remain to be discovered. Genomic technology is still improving in speed, cost and sophistication, and is likely to remain a fundamental tool of microbiology for many years to come.

## References

[1] Sabat AJ et al. 2013 Overview of molecular typing methods for outbreak detection and epidemiological surveillance. Euro Surveill. 18, 20380. 23369389 10.2807/ese.18.04.20380-en

[2] Maiden MC et al. 1998 Multilocus sequence typing: a portable approach to the identification of clones within populations of pathogenic microorganisms. Proc. Natl Acad. Sci. USA 95, 3140–3145. (10.1073/pnas.95.6.3140) 9501229 PMC19708

[3] Binnewies TT et al. 2006 Ten years of bacterial genome sequencing: comparative-genomics-based discoveries. Funct. Integr. Genomics 6, 165–185. (10.1007/s10142-006-0027-2) 16773396

[4] Fraser-Liggett CM 2005 Insights on biology and evolution from microbial genome sequencing. Genome Res. 15, 1603–1610. (10.1101/gr.3724205) 16339357

[5] Nierman W, Eisen JA, Fraser CM 2000 Microbial genome sequencing 2000: new insights into physiology, evolution and expression analysis. Res. Microbiol. 151, 79–84. (10.1016/S0923-2508(00)00125-X) 10865951

[6] Sanger F, Nicklen S, Coulson AR 1992 DNA sequencing with chain-terminating inhibitors. Biotechnology 24, 104–108. 1422003

[7] Quail MA, Smith ME, Coupland P, Otto TD, Harris SR, Connor TR, Bertoni A, Swerdlow HP, Gu Y 2012 A tale of three next generation sequencing platforms: comparison of ion torrent, Pacific biosciences and Illumina MiSeq sequencers. BMC Genomics 13, 341 (10.1186/1471-2164-13-341) 22827831 PMC3431227

[8] Hacker J, Kaper JB 2000 Pathogenicity islands and the evolution of microbes. Annu. Rev. Microbiol. 54, 641–679. (10.1146/annurev.micro.54.1.641) 11018140

[9] Perna NT et al. 2001 Genome sequence of enterohaemorrhagic *Escherichia coli* O157:H7. Nature 409, 529–533. (10.1038/35054089) 11206551

[10] Medini D, Donati C, Tettelin H, Masignani V, Rappuoli R 2005 The microbial pan-genome. Curr. Opin. Genet. Dev. 15, 589–594. (10.1016/j.gde.2005.09.006) 16185861

[11] Vernikos G, Medini D, Riley DR, Tettelin H 2015 Ten years of pan-genome analyses. Curr. Opin. Microbiol. 23C, 148–154. (10.1016/j.mib.2014.11.016) 25483351

[12] Parkhill J et al. 2001 Genome sequence of *Yersinia pestis*, the causative agent of plague. Nature 413, 523–527. (10.1038/35097083) 11586360

[13] Parkhill J et al. 2003 Comparative analysis of the genome sequences of *Bordetella pertussis*, *Bordetella parapertussis* and *Bordetella bronchiseptica*. Nat. Genet. 35, 32–40. (10.1038/ng1227) 12910271

[14] Parkhill J et al. 2001 Complete genome sequence of a multiple drug resistant *Salmonella enterica serovar* Typhi CT18. Nature 413, 848–852. (10.1038/35101607) 11677608

[15] Cummings CA, Brinig MM, Lepp PW, van de Pas S, Relman DA 2004 Bordetella species are distinguished by patterns of substantial gene loss and host adaptation. J. Bacteriol. 186, 1484–1492. (10.1128/JB.186.5.1484-1492.2004) 14973121 PMC344407

[16] Nierman WC et al. 2004 Structural flexibility in the *Burkholderia mallei* genome. Proc. Natl Acad. Sci. USA 101, 14 246–14 251. (10.1073/pnas.0403306101) 15377793 PMC521142

[17] Lee BM et al. 2005 The genome sequence of *Xanthomonas oryzae pathovar oryzae* KACC10331, the bacterial blight pathogen of rice. Nucleic Acids Res. 33, 577–586. (10.1093/nar/gki206) 15673718 PMC548351

[18] Mira A, Pushker R, Rodriguez-Valera F 2006 The Neolithic revolution of bacterial genomes. Trends Microbiol. 14, 200–206. (10.1016/j.tim.2006.03.001) 16569502

[19] Rasko DA et al. 2011 *Bacillus anthracis* comparative genome analysis in support of the Amerithrax investigation. Proc. Natl Acad. Sci. USA 108, 5027–5032. (10.1073/pnas.1016657108) 21383169 PMC3064363

[20] Ochman H, Elwyn S, Moran NA 1999 Calibrating bacterial evolution. Proc. Natl Acad. Sci. USA 96, 12 638–12 643. (10.1073/pnas.96.22.12638) PMC23026 10535975

[21] Ochman H, Wilson AC 1987 Evolution in bacteria: evidence for a universal substitution rate in cellular genomes. J. Mol. Evol. 26, 74–86. (10.1007/BF02111283) 3125340

[22] Harris SR et al. 2010 Evolution of MRSA during hospital transmission and intercontinental spread. Science 327, 469–474. (10.1126/science.1182395) 20093474 PMC2821690

[23] Croucher NJ et al. 2011 Rapid pneumococcal evolution in response to clinical interventions. Science 331, 430–434. (10.1126/science.1198545) 21273480 PMC3648787

[24] Mutreja A et al. 2011 Evidence for several waves of global transmission in the seventh cholera pandemic. Nature 477, 462–465. (10.1038/nature10392) 21866102 PMC3736323

[25] von Mentzer A et al. 2014 Identification of enterotoxigenic *Escherichia coli* (ETEC) clades with long-term global distribution. Nat. Genet. 46, 1321–1326. (10.1038/ng.3145) 25383970

[26] Sack DA, Sack RB, Nair GB, Siddique AK 2004 Cholera. Lancet 363, 223–233. (10.1016/S0140-6736(03)15328-7) 14738797

[27] Feng L et al. 2008 A recalibrated molecular clock and independent origins for the cholera pandemic clones. PLoS ONE 3, e4053 (10.1371/journal.pone.0004053) 19115014 PMC2605724

[28] Frerichs RR, Keim PS, Barrais R, Piarroux R 2012 Nepalese origin of cholera epidemic in Haiti. Clin. Microbiol. Infect. 18, E158–E163. (10.1111/j.1469-0691.2012.03841.x) 22510219

[29] Hendriksen RS et al. 2011 Population genetics of *Vibrio cholerae* from Nepal in 2010: evidence on the origin of the Haitian outbreak. mBio 2, e00157 (10.1128/mBio.00157-11) 21862630 PMC3163938

[30] Azarian T et al. 2014 Phylodynamic analysis of clinical and environmental *Vibrio cholerae* isolates from Haiti reveals diversification driven by positive selection. mBio 5, e01824 (10.1128/mBio.01824-14) 25538191 PMC4278535

[31] Eppinger M et al. 2014 Genomic epidemiology of the Haitian cholera outbreak: a single introduction followed by rapid, extensive, and continued spread characterized the onset of the epidemic. mBio 5, e01721 (10.1128/mBio.01721-14) 25370488 PMC4222100

[32] Katz LS et al. 2013 Evolutionary dynamics of *Vibrio cholerae* O1 following a single-source introduction to Haiti. mBio 4, e00398 (10.1128/mBio.00398-13) 23820394 PMC3705451

[33] Selezska K, Kazmierczak M, Müsken M, Garbe J, Schobert M, Häussler S, Wiehlmann L, Rohde C, Sikorski J 2012 *Pseudomonas aeruginosa* population structure revisited under environmental focus: impact of water quality and phage pressure. Environ. Microbiol. 14, 1952–1967. (10.1111/j.1462-2920.2012.02719.x) 22390474

[34] Wiehlmann L et al. 2007 Population structure of *Pseudomonas aeruginosa*. Proc. Natl Acad. Sci. USA 104, 8101–8106. (10.1073/pnas.0609213104) 17468398 PMC1876578

[35] Dettman JR, Rodrigue N, Aaron SD, Kassen R 2012 Evolutionary genomics of epidemic and nonepidemic strains of *Pseudomonas aeruginosa*. Proc. Natl Acad. Sci. USA 110, 21 065–21 070. (10.1073/pnas.1307862110) PMC3876195 24324153

[36] Drevinek P, Mahenthiralingam E 2010 *Burkholderia cenocepacia* in cystic fibrosis: epidemiology and molecular mechanisms of virulence. Clin. Microbiol. Infect. 16, 821–830. (10.1111/j.1469-0691.2010.03237.x) 20880411

[37] Bryant JM et al. 2013 Whole-genome sequencing to identify transmission of *Mycobacterium abscessus* between patients with cystic fibrosis: a retrospective cohort study. Lancet 381, 1551–1560. (10.1016/S0140-6736(13)60632-7) 23541540 PMC3664974

[38] McGee L et al. 2001 Nomenclature of major antimicrobial-resistant clones of *Streptococcus pneumoniae* defined by the pneumococcal molecular epidemiology network. J. Clin. Microbiol. 39, 2565–2571. (10.1128/JCM.39.7.2565-2571.2001) 11427569 PMC88185

[39] Munoz R et al. 1991 Intercontinental spread of a multiresistant clone of serotype 23F *Streptococcus pneumoniae*. J. Infect. Dis. 164, 302–306. (10.1093/infdis/164.2.302) 1856478

[40] Croucher NJ et al. 2014 Evidence for soft selective sweeps in the evolution of pneumococcal multidrug resistance and vaccine escape. Genome Biol. Evol. 6, 1589–1602. (10.1093/gbe/evu120) 24916661 PMC4122920

[41] Croucher NJ et al. 2014 Variable recombination dynamics during the emergence, transmission and ‘disarming’ of a multidrug-resistant pneumococcal clone. BMC Biol. 12, 49 (10.1186/1741-7007-12-49) 24957517 PMC4094930

[42] Croucher NJ et al. 2013 Dominant role of nucleotide substitution in the diversification of serotype 3 pneumococci over decades and during a single infection. PLoS Genet. 9, e1003868 (10.1371/journal.pgen.1003868) 24130509 PMC3794909

[43] Holden MT et al. 2013 A genomic portrait of the emergence, evolution, and global spread of a methicillin-resistant *Staphylococcus aureus* pandemic. Genome Res. 23, 653–664. (10.1101/gr.147710.112) 23299977 PMC3613582

[44] McAdam PR et al. 2012 Molecular tracing of the emergence, adaptation, and transmission of hospital-associated methicillin-resistant *Staphylococcus aureus*. Proc. Natl Acad. Sci. USA 109, 9107–9112. (10.1073/pnas.1202869109) 22586109 PMC3384211

[45] Ward MJ, Gibbons CL, McAdam PR, van Bunnik BAD, Girvan EK, Edwards GF, Fitzgerald JR, Woolhouse MEJ 2014 Time-scaled evolutionary analysis of the transmission and antibiotic resistance dynamics of *Staphylococcus aureus* CC398. Appl. Environ. Microbiol. 23, 7275–7282. (10.1128/AEM.01777-14) PMC4249192 25239891

[46] Brosch R et al. 2002 A new evolutionary scenario for the *Mycobacterium tuberculosis* complex. Proc. Natl Acad. Sci. USA 99, 3684–3689. (10.1073/pnas.052548299) 11891304 PMC122584

[47] Comas I et al. 2013 Out-of-Africa migration and Neolithic coexpansion of *Mycobacterium tuberculosis* with modern humans. Nat. Genet. 45, 1176–1182. (10.1038/ng.2744) 23995134 PMC3800747

[48] Bos KI et al. 2014 Pre-Columbian mycobacterial genomes reveal seals as a source of New World human tuberculosis. Nature 514, 494–497. (10.1038/nature13591) 25141181 PMC4550673

[49] Frank C et al. 2011 Epidemic profile of Shiga-toxin-producing *Escherichia coli* O104 : H4 outbreak in Germany. N. Engl. J. Med. 365, 1771–1780. (10.1056/NEJMoa1106483) 21696328

[50] Rohde H et al. 2011 Open-source genomic analysis of Shiga-toxin-producing *E. coli* O104 : H4. N. Engl. J. Med. 365, 718–724. (10.1056/NEJMoa1107643) 21793736

[51] Grad YH et al. 2013 Comparative genomics of recent Shiga toxin-producing *Escherichia coli* O104:H4: short-term evolution of an emerging pathogen. mBio 4, e00452-00412 (10.1128/mBio.00452-12) PMC3551546 23341549

[52] den Bakker HC et al. 2014 Rapid whole-genome sequencing for surveillance of *Salmonella enterica serovar enteritidis*. Emerg. Infect. Dis. 20, 1306–1314. (10.3201/eid2008.131399) 25062035 PMC4111163

[53] Lienau EK et al. 2011 Identification of a salmonellosis outbreak by means of molecular sequencing. N. Engl. J. Med. 364, 981–982. (10.1056/NEJMc1100443) 21345093

[54] Harris SR et al. 2013 Whole-genome sequencing for analysis of an outbreak of meticillin-resistant *Staphylococcus aureus*: a descriptive study. Lancet 13, 130–136. (10.1016/S1473-3099(12)70268-2) 23158674 PMC3556525

[55] Snitkin ES, Zelazny AM, Thomas PJ, Stock F, Henderson DK, Palmore TN, Segre JA, NISC Comparative Sequencing Program. 2012 Tracking a hospital outbreak of carbapenem-resistant *Klebsiella pneumoniae* with whole-genome sequencing. Sci. Transl. Med. 4, 148ra116 (10.1126/scitranslmed.3004129) PMC3521604 22914622

[56] Halachev MR, Chan J, Constantinidou CI, Cumley N, Bradley C, Smith-Banks M, Oppenheim B, Pallen MJ 2014 Genomic epidemiology of a protracted hospital outbreak caused by multidrug-resistant *Acinetobacter baumannii* in Birmingham, England. Genome Med. 6, 70 (10.1186/s13073-014-0070-x) 25414729 PMC4237759

[57] Worby CJ, Lipsitch M, Hanage WP 2014 Within-host bacterial diversity hinders accurate reconstruction of transmission networks from genomic distance data. PLoS Comput. Biol. 10, e1003549 (10.1371/journal.pcbi.1003549) 24675511 PMC3967931

[58] Golubchik T et al. 2013 Within-host evolution of *Staphylococcus aureus* during asymptomatic carriage. PLoS ONE 8, e61319 (10.1371/journal.pone.0061319) 23658690 PMC3641031

[59] Tong SY et al. 2014 Genome sequencing defines phylogeny and spread of methicillin-resistant *Staphylococcus aureus* in a high transmission setting. Genome Res. 25, 111–118. (10.1101/gr.174730.114) 25491771 PMC4317166

[60] Eyre DW et al. 2013 Diverse sources of *C. difficile* infection identified on whole-genome sequencing. N. Engl. J. Med. 369, 1195–1205. (10.1056/NEJMoa1216064) 24066741 PMC3868928

[61] Walker TM et al. 2013 Whole-genome sequencing to delineate *Mycobacterium tuberculosis* outbreaks: a retrospective observational study. Lancet 13, 137–146. (10.1016/S1473-3099(12)70277-3) 23158499 PMC3556524

[62] Bryant JM et al. 2013 Whole-genome sequencing to establish relapse or re-infection with *Mycobacterium tuberculosis*: a retrospective observational study. Lancet 1, 786–792. (10.1016/S2213-2600(13)70231-5) 24461758 PMC3861685

[63] Guerra-Assuncao JA et al. 2014 Recurrence due to relapse or reinfection with *Mycobacterium tuberculosis*: a whole-genome sequencing approach in a large, population-based cohort with a high HIV infection prevalence and active follow-up. J. Infect. Dis. 211, 1154–1163. (10.1093/infdis/jiu574) 25336729 PMC4354982

[64] Gardy JL et al. 2011 Whole-genome sequencing and social-network analysis of a tuberculosis outbreak. N. Engl. J. Med. 364, 730–739. (10.1056/NEJMoa1003176) 21345102

[65] Marston BJ, Lipman HB, Breiman RF 1994 Surveillance for Legionnaires’ disease: risk factors for morbidity and mortality. Arch. Intern. Med. 154, 2417–2422. (10.1001/archinte.1994.00420210049006) 7979837

[66] Bull M, Hall IM, Leach S, Robesyn E 2012 The application of geographic information systems and spatial data during Legionnaires disease outbreak responses. Euro Surveill. 17, 20331. 23231895 10.2807/ese.17.49.20331-en

[67] McAdam PR et al. 2014 Gene flow in environmental *Legionella pneumophila* leads to genetic and pathogenic heterogeneity within a Legionnaires’ disease outbreak. Genome Biol. 15, 504 (10.1186/PREACCEPT-1675723368141690) 25370747 PMC4256819

[68] Johansson A et al. 2014 An outbreak of respiratory tularemia caused by diverse clones of *Francisella tularensis*. Clin. Infect. Dis. 59, 1546–1553. (10.1093/cid/ciu621) 25097081 PMC4650766

[69] Didelot X, Bowden R, Wilson DJ, Peto TE, Crook DW 2012 Transforming clinical microbiology with bacterial genome sequencing. Nat. Rev. Genet. 13, 601–612. (10.1038/nrg3226) 22868263 PMC5049685

[70] Koser CU et al. 2012 Routine use of microbial whole genome sequencing in diagnostic and public health microbiology. PLoS Pathog. 8, e1002824 (10.1371/journal.ppat.1002824) 22876174 PMC3410874

[71] Chewapreecha C et al. 2014 Comprehensive identification of single nucleotide polymorphisms associated with beta-lactam resistance within pneumococcal mosaic genes. PLoS Genet. 10, e1004547 (10.1371/journal.pgen.1004547) 25101644 PMC4125147

[72] Farhat MR et al. 2013 Genomic analysis identifies targets of convergent positive selection in drug-resistant *Mycobacterium tuberculosis*. Nat. Genet. 45, 1183–1189. (10.1038/ng.2747) 23995135 PMC3887553

[73] Sheppard SK et al. 2013 Genome-wide association study identifies vitamin B5 biosynthesis as a host specificity factor in *Campylobacter*. Proc. Natl Acad. Sci. USA 110, 11 923–11 927. (10.1073/pnas.1305559110) PMC3718156 23818615

[74] Ashton PM, Nair S, Dallman T, Rubino S, Rabsch W, Mwaigwisya S, Wain J, O'Grady J 2014 MinION nanopore sequencing identifies the position and structure of a bacterial antibiotic resistance island. Nat. Biotechnol. 33, 296–300. (10.1038/nbt.3103) 25485618

[75] Plucain J et al. 2014 Epistasis and allele specificity in the emergence of a stable polymorphism in *Escherichia coli*. Science 343, 1366–1369. (10.1126/science.1248688) 24603152

[76] Manso AS et al. 2014 A random six-phase switch regulates pneumococcal virulence via global epigenetic changes. Nat. Commun. 5, 5055 (10.1038/ncomms6055) 25268848 PMC4190663

